# Outcome measures reported by cancer patients treated with tyrosine kinase inhibitors: a methodological study

**DOI:** 10.1590/0034-7167-2024-0018

**Published:** 2025-03-31

**Authors:** Ana Maria Teixeira Pires, Julie Ann Ponto, Eliana Cavalari Teraoka, Fabio Rodrigues Kerbauy, Edvane Birelo Lopes De Domenico

**Affiliations:** I Universidade Federal de São Paulo. São Paulo, São Paulo, Brazil; II Winona State University. Rochester, Minessota, United States of America

**Keywords:** Patient Reported Outcome Measures, Protein-Tyrosine Kinases, Signs and Symptoms, Patient-Centered Care, Validation Study., Medición de Resultados Informados por el Paciente, Proteínas Tirosina Quinasas, Signos y Síntomas, Atención Dirigida al Paciente, Estudio de Validación.

## Abstract

**Objectives:**

to validate the content of an outcome measurement instrument with expert judges and to assess the understandability and suitability of this validated instrument with cancer patients undergoing tyrosine kinase inhibitor (TKI) therapy.

**Methods:**

a methodological study, which included the development of an instrument through integrative review, content validity, using the Delphi technique with expert judges, and pilot testing, to verify users’ understandability and suitability to the instrument.

**Results:**

literature review allowed constructing PRO-CTCAE^®^ Estudo PROM-TKI Brasil, with 16 items, which was later submitted to validity using the Delphi technique. After the second round, the final instrument consisted of 20 items, with a Content Validity Index (CVI) of 0.878. The pilot test showed that the instrument is understandable and suitable for the target population (CVI of 1.0).

**Conclusions:**

PRO-CTCAE^®^ Estudo PROM-TKI Brasil obtained evidence of validity for use during TKI therapy.

## RESUMEN

**Objetivos:**

validar el contenido de un instrumento de medición de resultados con jueces expertos y evaluar la comprensibilidad e idoneidad de este instrumento validado con pacientes con cáncer en tratamiento con inhibidores de la tirosina quinasa (TKI).

**Métodos:**

estudio metodológico, que incluyó el desarrollo de un instrumento mediante revisión integradora, validación de contenido, utilizando la técnica Delphi con jueces expertos y pruebas piloto, para verificar la comprensibilidad e idoneidad del instrumento por parte de los usuarios.

**Resultados:**

la revisión de la literatura permitió la construcción del PRO-CTCAE^®^ Estudo PROM-TKI Brasil, con 16 ítems, posteriormente sometido a validación mediante la técnica Delphi. Luego de la segunda ronda, el instrumento final estuvo conformado por 20 ítems, con un Índice de Validez de Contenido (IVC) de 0,878. La prueba piloto demostró que el instrumento es comprensible y adecuado para la población objetivo (CVI de 1,0).

**Conclusiones:**

el PRO-CTCAE^®^ Estudo PROM-TKI Brasil obtuvo evidencia de validación para su uso durante la terapia con TKI.

## INTRODUCTION

Cancer remains a global challenge, with an estimated 19.3 million new cases (18.1 million excluding non-melanoma skin cancer) and nearly 10.0 million deaths (9.9 million excluding non-melanoma skin cancer), according to 2020 data^(1)^.

However, the fight against cancer through drug therapy has gained strong allies, such as targeted therapies, which have specific cell receptors and markers as their main means of combat. The isolated use of these new drugs or in combination with cytotoxic chemotherapeutics has resulted in better prognoses for patients with different types of cancer^(2)^.

Among the advances, studies of cancer biology have made it possible to understand the molecular structures and signaling pathways of tyrosine kinase receptors, which have generated several drugs then called protein tyrosine kinase inhibitors (TKIs)^(3)^. These drugs are generally well tolerated, tend to have a predictable toxicity profile and are less severe than cytotoxic chemotherapy, which does not mean that it is not necessary to manage their effects and avoid aggravated conditions that indicate significant impairment of functionality or the need for emergency intervention (normally classified as grades 3 and 4, respectively), occasions that inevitably lead to treatment interruption and, therefore, a worse chance of a good outcome^(4)^.

Recognizing adverse effects (AEs) is the first stage towards adopting preventive and therapeutic approaches. Within the premise of providing quality and patient safety, tools are implemented to provide support for clinical practice, characterizing AEs and identifying interventions capable of producing safe and effective actions. However, invariably, healthcare professionals are the ones who make an estimate of the severity of AEs reported by patients, and this is not always true to reality^(5)^.

Certainly, the ability to self-monitor conditions related to signs and symptoms needs to be developed in patients, and facilitating instruments should be adapted to each country’s culture. Tools such as Patient Reported Outcome Measures (PROMs) allow patients to reflect on their condition and define what is important from their perspective, encouraging them to discuss their problems and improving communication with healthcare professionals^(6)^.

The PRO-CTCAE™ scale, Patient Reported Outcome (PRO) - Common Terminology Criteria for Adverse Events (CTCAE), is a PROM tool model from the National Cancer Institute, United States of America, composed of 78 items, encompassing all possible symptoms for the oncological therapeutic spectrum^(6)^. Its creation was based on reading all CTCAE items that could be reported by patients. Then, specific descriptors for each item were selected based on other questionnaires used in oncology, which included degrees of measurement, such as symptom severity, frequency and its interference with quality of life, with a score of 0 to 4 on a 5-point Likert scale (none, mild, moderate, severe and very severe), including items such as presence/absence, frequency, severity and interference with daily activities.

A patient is asked to respond, with the guidance “please select the one response that best describes your experiences over the past 7 days”^(6)^. From there, the symptoms were added to one, two or three of these questions. Construct, reliability and understandability validity was successful^(6)^.

PRO-CTCAE™ allows us to not only assess patients’ signs and symptoms, but also to make them actively participate in their care at a time when a patient experience has been so valued and studied^(6)^.

A professional can choose, beforehand or during therapy, which toxicities patients are susceptible to and search for correspondence in PRO-CTCAE™, and must also choose the frequency and time to start collecting this information. PRO-CTCAE™ development and validity have been consistent and well established by the US Food and Drug Administration (FDA) and the European Medicines Agency (EMA)^(6)^. The process included cancer patients, professionals with expertise in oncology, development instruments, clinical research and regulatory aspects.

Validity, reliability and psychometric tests were performed in English and Spanish. Data can be obtained via the web, an interactive voice response system and printed papers, offering flexibility to choose the best option for the population studied. The computerized system can include alerts and alarms for severe symptoms^(6)^, directing them to the care team, either through telemedicine or by visiting the original department or the emergency room in person.

Using PRO-CTCAE™ in its entirety in healthcare practice is not feasible due to the number of symptoms it encompasses. Therefore, considering the need for effective monitoring and care focused on cancer patients using TKI drugs, a complex intervention study was designed, as proposed by the UK Medical Research Council (MRC)^(7)^. The stages presented in this article are all those that precede the clinical trial itself. The first stage was a literature review, followed by the content validity and piloting stages. The guiding questions were: what AEs do TKI drugs cause in patients? How do patients assess the understandability and suitability of a PROM-type instrument specific to symptoms caused by TKI drugs?

## OBJECTIVES

To validate the content of *PRO-CTCAE^®^ Estudo PROM-TKI Brasil* items with expert judges and to assess the understandability and suitability of this validated instrument with cancer patients undergoing TKI therapy.

## METHODS

### Ethical aspects

The study was approved by the *Universidade Federal de São Paulo* (UNIFESP) and the *Hospital Alemão Oswaldo Cruz* Research Ethics Committees. The study is registered in the Brazilian Clinical Trials Registry (https://ensaiosclinicos.gov.br/).

### Study design, period and site

This is a methodological study, developed in four stages: integrative review; instrument development based on the review; content validity of the new instrument items using the Delphi technique with experts; and pilot testing with the target audience to assess instrument suitability and understandability (stages completed and described in this article)^(8)^. Each stage of study was based on specific methodological procedures, and to ensure the quality of the description of the research stages, the study used the Standards for Quality Improvement Reporting Excellence (SQUIRE) 2.0 checklist^(9)^.

The study began in March 2021, in its first phase of execution, and continued until pilot testing, completed in September 2022. Pilot testing was multicenter, in which two general hospitals participated, one private and one public, in the city of São Paulo, São Paulo, Brazil, and which have oncology care centers, including outpatient units.

### Study protocols

The study protocols concerning each stage are detailed in the sequence of occurrence.

### Stage 1: Integrative review

The integrative literature review provided the search, critical assessment and synthesis of available evidence on the topic investigated^(10)^. To guide the search for scientific articles, the SPIDER strategy was used, as it is more suitable for studies of a mixed and/or exclusively qualitative nature and because it fits the research objectives^(11)^. The elements that made up the acronym SPIDER are found in Chart 1 and allowed formulating the study question: which AEs of TKIs (PI and E) in cancer patients (S) are reported in literature (D and R)?

**Chart 1:** Descriptors selected by the SPIDER technique

SPIDER	Descriptors
Sample	“oncology patients”
Phenomenon of Interest	“protein kinase inhibitor” OR “tyrosine kinase inhibitor” AND “adverse effects” OR “adverse drug reaction”
Design	“meta-analysis” OR “systematic review” OR “review”
Evaluation	“adverse effects” OR “adverse drug reaction” AND “tyrosine kinase inhibitor”
Research type	“quantitative” OR “qualitative”, “mixed methods”

This study included only literature reviews, whether integrative, systematic with or without meta-analysis, available in full, in Portuguese, Spanish and English, from 2018 to February 2021, in the National Library of Medicine (PubMed), Web of Science and Embase databases.

**Figure 1:**
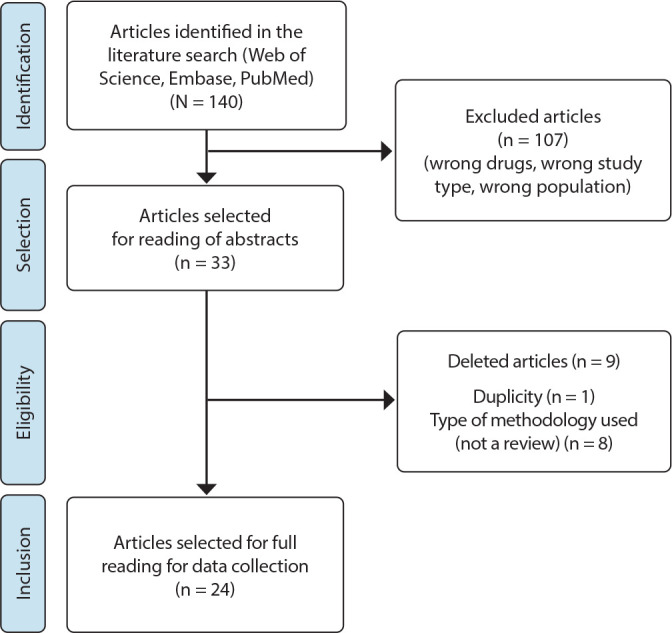
Flowchart of the PRISMA model for article selection, 2021

A total of 140 articles were selected from the three databases following the Preferred Reporting Items for Systematic Reviews and Meta-Analyses (PRISMA) criteria (Figure 1). These articles were transferred to the Rayyan application^(12)^. This application allows each title to be analyzed, identified as included or not, detailed the reason for exclusion and made small notes. These actions facilitated the final count and recording of all phases of the method used.

After reading the titles, 107 articles were excluded, and the main reasons were that they addressed other drugs, non-oncological pathologies and other research methods. After reading the 33 abstracts, eight were excluded because they did not answer the study questions and one was duplicated. The remaining 24 articles that met the inclusion criteria comprised the final sample. The article was read in full to assess the study design and look for data of interest to the research, selecting the sentences that described the AEs of TKIs^(13-36)^. All AEs were listed and organized into groups for subsequent item search in the original PRO-CTCAE™, except those that only appeared in one article (blurred vision, corneal injury, blepharitis, dry eyes, voice change).

### Stage 2 - *PRO-CTCAE^®^ Estudo PROM-TKI Brasil* development

For each AE found more than once in the integrative review, a similar item was sought in the original PRO-CTCAE™. Therefore, 16 items from the document plus an open-ended question, also included in the original, were chosen to create the *PRO-CTCAE^®^ Estudo PROM-TKI Brasil* version.

### Stage 3 - *PRO-CTCAE^®^ Estudo PROM-TKI Brasil* content validity

Validity is a critical factor in an instrument selection and application in practice and research, and means the extent to which the instrument measures what it is intended to measure^(37,38)^. For instance, to assess patient health status, the Food and Drug Administration (FDA) provides guidance for assessing and reviewing PRO instruments used to measure treatment, benefit, or risk associated with medical products, based on their conceptual framework and content validity. The FDA encourages instrument developers to provide evidence of the instrument’s content using literature review and opinions from experts and target patients^(37,38)^.

Content validity involves the participation of subject matter experts to assess content relevance and the extent to which each instrument item proves the phenomenon of interest and the dimension of each item in the set of what is intended to be assessed^(38)^.

The Delphi technique allows for gathering expert opinions, without restrictions on geographical areas, on varied and complex topics^(39)^. In practice, experts respond to a structured questionnaire anonymously in rounds. The data from the first round are analyzed by the researcher, and agreement is calculated quantitatively. Subsequently, the summary of modifications, the agreement quantitative analysis and the reformulated document are sent to the same experts, forming the 2^nd^ round and so on. The rounds are concluded based on consensus among specialists, also called judges or experts^(39)^.

### Site, sample, inclusion and exclusion criteria

Oncologists and nurses specializing in oncology who are part of both institutions participating in the study were invited.

Oncologists and nurses who scored 4 points or more in Fehring’s adapted classification were chosen, which consists of a specialist’s score according to their professional performance, qualification and production of knowledge in the field of knowledge investigated^(40)^. Judges’ field of expertise considered experience with patients undergoing TKI therapy in both research and daily care. Physicians had to be prescribers and responsible for the care of these patients, and nurses had to work in practice with this therapy. The exclusion criterion consisted of partial completion of one of the data collection instruments.

### Procedures for data collection

Participant selection was carried out by individualized email message, clarification of doubts, acceptance of participation, signing of the Informed Consent Form (ICF), according to recommendations of Resolution 466/12 of the Brazilian National Health Council (In Portuguese, *Conselho Nacional de Saúde* - CNS), and, subsequently, the data were collected.

Two structured instruments were used: the first with personal identification (sex and age) and professional activity, and the second was specific for content validity. There was no nominal identification of respondents in the document, being coded with sequential numbers. *PRO-CTCAE^®^ Estudo PROM-TKI Brasil* was sent and clarified regarding its construction. Each item of the instrument (symptom) was assessed based on criteria considered appropriate for the purpose of the investigation, such as objectivity, simplicity, clarity, relevance and precision^(38)^. For each item, the judge marked one of the alternatives on the Likert scale: 1: totally disagree; 2: disagree; 3: agree; 4: totally agree. At the end of each item, there was the possibility of inserting an observation and, at the end of all items, there was the question “Is there any other symptom that you think should be included? If so, which one?”, which generated the possibility of suggesting other symptoms not presented and that they considered to be important in clinical management of patients undergoing oral therapy with TKI.

To analyze the process of each Delphi round, the Content Validity Index (CVI) was calculated. The minimum desirable agreement was 0.80^(41)^.

### Stage 4 - Pilot testing

The instrument constructed after literature review and validity with expert judges was used for pilot testing to assess instrument understandability and suitability after content validity, now by end users, patients using oral TKI drugs. To assess the proposed objectives, a small sample of participants may generate the desired responses or use the percentage of 10% of the study sample in the next phase^(42)^. The sample size for the next phase will be for convenience, based on recommendations that consider that, for a 5% level of significance, it is desirable to include five to ten individuals per questionnaire item^(43)^. As the questionnaire contains 20 items, the sample will be 100 to 200 patients; therefore, in this pilot, ten to 20 patients are recommended.

Pilot testing was designed to assess the following hypotheses:

H1: *PRO-CTCAE^®^ Estudo PROM-TKI Brasil* is easy to understand and easy to complete;

H2: *PRO-CTCAE^®^ Estudo PROM-TKI Brasil* includes symptoms presented by patients.

### Location, sample, inclusion and exclusion criteria

Patients from both institutions were randomly invited on the days of outpatient medical appointments, constituting a convenience sample.

Patients over 18 years of age, diagnosed with kidney or lung tumor or chronic myeloid leukemia who had been undergoing treatment with any TKI for more than two months and with clinical functionality assessed by the Eastern Cooperative Oncologic Group (ECOG) scale between 0 and 2 were included. Patients with multiple comorbidities (≥ 3) and/or who partially completed one of the data collection instruments were excluded.

### Procedures for data collection

After selecting participants, a personal contact was made on the day of the medical appointment using a pre-established script, inviting them to participate. At this time, information was provided and, after signing the ICF, the instruments were applied. The participant characterization instrument consisted of sociodemographic and clinical data (age, sex, diagnosis of oncological disease, pre-existing disease and medications in use).

Participants were instructed to respond to the *PRO-CTCAE^®^ Estudo PROM-TKI Brasil* validated in the previous stage, presented in printed form, after signing the ICF. For each symptom item in the instrument, there were two statements: “I can understand what is written” and “I can self-assess and choose the alternative that indicates how I am”. The alternatives for each statement were on a 4-point Likert scale: 1: completely disagree; 2: disagree; 3: agree; 4: completely agree. Hence, they were asked about font size and type and presentation format in a booklet, accompanied by the alternatives above. Finally, a space was left for writing suggestions.

Sociodemographic data were assessed descriptively, using absolute numbers and percentages, and for Likert-type scale responses, CVI was calculated, which indicates the percentage of agreement among respondents. The minimum desirable agreement was 0.80^(41)^. SPSS Statistics version 28.0 (IBM Corp., Armonk, NY, USA) was used. Field describes in detail the theoretical basis used for the data analysis presented in this report^(44)^.

## RESULTS

In the literature review stage, 24 articles were selected, and, in these articles, 58 AEs were found. Subsequently, it was decided to organize AEs into eight groups. The groups were named as gastrointestinal, dermatological, cardiac, renal, pulmonary, myelotoxicity, fatigue and others (effects that did not fit into the previous items).

Of the gastrointestinal AEs, nausea, vomiting, decreased appetite, diarrhea, constipation and mucositis were symptoms found in PRO-CTCAE™, with mucositis being related to “dry mouth” and “mouth/throat sores”. Weight loss was an AE found, but as it is a signal that requires measurement, it is not answered subjectively and, therefore, has no analogue in PRO-CTCAE™.

Regarding the integumentary system, the effects found were rash, paronychia, pruritus, hand-foot syndrome and xerosis. In PRO-CTCAE™, skin dryness, itching and hand-foot syndrome were found and were included. Concerning paronychia, PRO-CTCAE™ contains three items related to the nail that are conceptually different (if there was any nail loss, nail ridging and nail discoloration) and, therefore, were not considered.

Hepatotoxicity and changes in renal or cardiac function are signs obtained through laboratory tests that are not perceived by patients, and therefore have no analogues in PRO-CTCAE™. Hypertension, for instance, requires measurement, just as an electrocardiogram is necessary to observe cardiac changes. However, shortness of breath may indicate a lung change and is therefore included in the instrument.

Only one article cited edema as an AE, but without specification. In PRO-CTCAE™, there are three items on edema in specific locations (swelling, bloating, and breast swelling and tenderness) that were not considered^(13-36)^, because the article citation does not specify the edema site, contrary to what is found in PRO-CTCAE™. Including the three items on swelling would increase the number of items to a quantity that might not be acceptable. Favoring this initiative, in the second round, when bloating was included (at the suggestion of a judge in the first round), none of the three judges approved it, and the item swelling was accepted by only one judge, with a CVI of 0.33, which invalidates its inclusion.

As for myelotoxicity, since it is an effect verified through laboratory tests, it has no analogue in PRO-CTCAE™. Fever could be a perceived symptom, but it is mainly defined through temperature measurement, and has no analogue in PRO-CTCAE™. Fatigue was an AE found in literature and is present in PRO-CTCAE™, which has been included^(13-36)^.

Some metabolic alterations, such as hyperglycemia, hypercholesterolemia, hypothyroidism and hypocalcemia, were found in literature, but they are not symptoms perceived by patients, and have no analogue in PRO-CTCAE™.

In relation to ocular changes, only one article detailed symptoms such as dry eye, conjunctivitis, cataracts and corneal injury, which were not included according to established exclusion criteria^(13-36)^.

Each AE found in the same terms in PRO-CTCAE™ was transferred to a new document, comprising 16 items. Since the last question (17^th^) referred to any symptom that patients could experience, the instrument would be able to encourage patients to remember some other symptom not covered and respond about it, since the last questions consisted of: do you have any other symptoms that you wish to report? Which one? In the last 7 days, what was the severity of this symptom at its worst? The new document, from then on, was called *PRO-CTCAE^®^ Estudo PROM-TKI Brasil*.

### *PRO-CTCAE^®^ Estudo PROM-TKI Brasil* content validity

The completion of stage 3 of this study (instrument validity through consensus of expert judges) added veracity to item selection, since, in addition to using data from a literature review, judges’ knowledge and practice were considered. The first criterion for choosing judges was being a professional from either of the two institutions in the study. The second criterion followed Fehring’s adapted classification, which resulted in an experienced group of judges.

In the validity process, *PRO-CTCAE^®^ Estudo PROM-TKI Brasil* was sent to 15 judges (13 oncologists and two nurses) individually by email, along with an explanation about the research, its importance and objectives, requesting a response and establishing a deadline of 15 days. After seven days, another email was sent as a reminder to each judge. After this time elapsed, a new email was sent accepting a deadline of another seven days. In the first round, six completed ICFs and six answered questionnaires were received, corresponding to the sample of the first round. Judges were composed of five oncologists (one PhD, two masters and two specialists with more than ten years of activity in the field) and one nurse (a masters with more than ten years of activity in the field).

In the first round, CVI was 0.878, showing that the choice of items could be maintained. Through an open-ended question about suggestions for inclusions, responding judges suggested five symptoms, which were included to be submitted for validity in the second round. The Delphi technique therefore allowed, through its rounds, improving the document based on judges’ expertise. Once this was done, the data were analyzed, and the three items that achieved a CVI of 0.67 (although lower than 0.8) were included in the questionnaire, which had a total of 20 questions, a reasonable number for patient acceptance^(45-48)^.

Table 1 presents the CVI values for each calculated item contained in the initial *PRO-CTCAE^®^ Estudo PROM-TKI Brasil* ^(49)^.

**Table 1:** Content Validity Index according to the question and according to the type of equivalence for *PRO-CTCAE^®^ Estudo PROM-TKI Brasil*

Item	Criterion					Mean per question
	Objectivity	Simplicity	Clarity	Relevance	Precision	
Severity of dry mouth^*^	0.83	0.67	0.67	0.83	0.83	0.77
Severity of mouth/throat sores	1.00	0.83	0.83	1.00	0.83	0.90
Interference of mouth/throat sores with daily activities	1.00	1.00	0.83	1.00	0.83	0.93
Severity of decreased appetite	1.00	0.83	0.83	1.00	0.83	0.90
Interference of decreased appetite with daily activities^*^	0.67	0.67	0.33	0.83	0.50	0.60
Frequency of nausea	1.00	1.00	1.00	1.00	1.00	1.00
Severity of nausea^*^	0.83	0.83	0.67	1.00	1.00	0.87
Frequency of vomiting^*^	0.83	0.83	0.67	1.00	1.00	0.87
Severity of vomiting^*^	0.83	0.67	0.67	0.83	0.833	0.77
Severity of constipation^*^	1.00	0.83	0.667	1.00	1	0.90
Frequency of diarrhea	0.83	0.83	0.83	0.83	0.83	0.83
Severity of shortness of breath	1.00	0.83	0.833	1.00	1.00	0.93
Interference of shortness of breath with daily activities^*^	1.00	0.83	0.50	1.00	0.83	0.83
Severity of cough^*^	0.83	0.67	0.67	0.83	0.83	0.77
Interference of cough with daily activities^*^	0.83	0.83	0.67	0.83	0.83	0.80
Severity of skin dryness	1.00	0.83	0.83	0.83	1.00	0.90
Severity of itching	1.00	0.83	0.83	1.00	1.00	0.93
Severity of hand-foot syndrome^*^	0.83	0.83	0.67	1.00	0.83	0.83
Severity of numbness or tingling in the hands and feet	1.00	0.83	0.83	1.00	1.00	0.93
Interference of numbness or tingling with daily activities	1.00	0.83	0.83	1.00	1.00	0.93
Frequency of general pain	1.00	1.00	1.00	1.00	1.00	1.00
Severity of general pain	1.00	0.83	0.83	1.00	1.00	0.93
Interference of general pain with daily activities	1.00	0.83	0.83	1.00	1.00	0.93
Severity of fatigue	1.00	0.83	0.83	1.00	1.00	0.93
Interference of fatigue with daily activities	1.00	0.83	0.83	1.00	1.00	0.93
Presence of bruising	1.00	1.00	1.00	0.83	1.00	0.97
Other symptoms^*^	0.83	0.67	0.67	1.00	0.83	0.80
Mean per criterion	0.93	0.83	0.76	0.95	0.91	0.88

Table 1 results demonstrated that, as a whole, *PRO-CTCAE^®^ Estudo PROM-TKI Brasil* achieved the minimum CVI (0.878) proposed by Davis^(50)^, but did not reach the recommended CVI (≥ 0.9) suggested by Lynn^(51)^ and Waltz^(49)^. In relation to each criterion, it was observed that clarity was below 0.8 and simplicity was between 0.8 and 0.899, whereas the other criteria had a value greater than or equal to 0.9.

Regarding the CVI for each item, the following items did not reach the minimum CVI of 0.78 proposed by Lynn^(51)^ for samples of six to ten judges (marked with an asterisk*): dry mouth: simplicity and clarity; interference of decreased appetite with daily activities: objectivity, simplicity, clarity and precision; severity of nausea: clarity; frequency of vomiting: clarity; severity of vomiting: simplicity and clarity; severity of constipation: clarity; interference of shortness of breath with daily activities: clarity; severity of cough: simplicity and clarity; interference of cough with daily activities: clarity; severity of hand-foot syndrome: clarity; other symptoms: simplicity and clarity. Although the clarity criterion achieved the lowest index (0.76), the pilot test showed excellent understandability (CVI = 1) by the target population. The other items achieved the minimum CVI in their respective criteria.

Additionally, in the open-ended question, participants suggested changes to the wording of some items present in PRO-CTCAE™ that would modify the version made available in Portuguese, and these were not accepted, as there are rules of use that restrict any modification^(52)^. However, there were also suggestions of signs and symptoms that were not included in the instrument, such as insomnia, bloating, swelling, acne and hives.

After the researchers carefully read the original version of PRO-CTCAE™, they found analogs to the suggested signs and symptoms. Thus, the version for the second round of *PRO-CTCAE^®^ Estudo PROM-TKI Brasil* was created with the aim of assessing agreement regarding the new items. This version was sent to expert judges, together with explanations of the changes made and not made, and three participants responded. Table 2 shows the results of the second round, with the CVI values for each item.

**Table 2:** Content Validity Index of symptoms assessed in the second round according to the item for incorporation into *PRO-CTCAE^®^ Estudo PROM-TKI Brasil*

Item	CVI
Cracking at the corners of the mouth (cheilosis/cheilitis)	0.67
Acne	0.67
Hives	0.67
Insomnia	0.33
Swelling	0.33
Bloating	0.00

Although CVI did not show total agreement (0.67), the symptoms “cracking at the corners of the mouth (cheilosis/cheilitis)”, “acne” and “hives” were included, corresponding to the dermatological group, in accordance with suggestion of specialized literature that advocates for majority agreement^(51)^. The symptoms excluded were “bloating” and “insomnia”, as they were accepted by only one judge (CVI: 0.33), and “swelling”, as it was not accepted at all (CVI: 0.0).

The final version of *PRO-CTCAE^®^ Estudo PROM-TKI Brasil* is shown to be suitable for patients undergoing this treatment modality.

### Instrument assessment for understandability and suitability

The pilot test population consisted of 18 patients from the institutions participating in the study. The majority (n: 12) were male (66.7%); ten were Brazilian Health System users (55.6%); eight were supplementary health users (44.4%); four were single (22.2%); 12 were married (66.7%); two were widowed (11.1%); one was a student (6.2%); 11 were employed (68.7%); four were retired (25.0%) (two did not respond). As for the level of education, one had incomplete elementary education (5.6%); one had complete elementary education (5.6%); eight had complete medical education (44.4%); eight had complete higher education (4.4%). In clinical functionality characterization, 15 patients had ECOG (PS) = 0 (83.3%), and three had Performance Status (PS) = 1 (16.7%). Regarding comorbidities, five patients (27.8%) had isolated or accumulated comorbidities of diabetes, hypertension, hypercholesterolemia and hypothyroidism.

Concerning diagnosis and treatment, ten patients were diagnosed with chronic myeloid leukemia (55.6%); four patients were diagnosed with kidney cancer (22.2%); three patients were diagnosed with lung cancer (16.7%); and one patient was diagnosed with Hodgkin’s lymphoma (5.6%). The majority (n: 13) received TKI alone (72.2%), and five patients received TKI associated with another antineoplastic medication (27.8%). The TKI drugs mentioned by patients were imatinib, nilotinib, axitinib, ibrutinib, osimertinib and alectinib.

Table 3 shows the frequencies of responses indicated by participants for each item of *PRO-CTCAE^®^ Estudo PROM-TKI Brasil*.

**Table 3:** Sample distribution in relation to responses per item in *PRO-CTCAE^®^ Estudo PROM-TKI Brasil*

PRO-CTCAE® Estudo PROM-TKI Brasil questions	Did not answer		None		Mild		Moderate		Severe		Very severe	
	n	%	n	%	n	%	n	%	n	%	n	%
Severity of dry mouth	0	0.00	10	55.56	5	27.78	2	11.11	0	0.00	1	5.56
Severity of mouth/throat sores	0	0.00	17	94.44	0	0.00	1	5.56	0	0.00	0	0.00
Interference of mouth/throat sores with daily activities	2	11.11	16	88.89	0	0.00	0	0.00	0	0.00	0	0.00
Severity of cracking at the corners of the mouth (cheilosis/cheilitis)	0	0.00	17	94.44	1	5.56	0	0.00	0	0.00	0	0.00
Severity of decreased appetite	0	0.00	16	88.89	2	11.11	0	0.00	0	0.00	0	0.00
Interference of decreased appetite with daily activities	1	5.56	17	94.44	0	0.00	0	0.00	0	0.00	0	0.00
Frequency of nausea	0	0.00	13	72.22	1	5.56	3	16.67	1	5.56	0	0.00
Severity of nausea	1	5.56	12	66.67	4	22.22	1	5.56	0	0.00	0	0.00
Frequency of vomiting	0	0.00	16	88.89	0	0.00	2	11.11	0	0.00	0	0.00
Severity of vomiting	1	5.56	15	83.33	1	5.56	1	5.56	0	0.00	0	0.00
Severity of constipation	0	0.00	12	66.67	4	22.22	2	11.11	0	0.00	0	0.00
Frequency of diarrhea	0	0.00	14	77.78	0	0.00	1	5.56	1	5.56	2	11.11
Severity of shortness of breath	0	0.00	15	83.33	1	5.56	1	5.56	1	5.56	0	0.00
Interference of shortness of breath with daily activities	1	5.56	16	88.89	0	0.00	0	0.00	1	5.56	0	0.00
Severity of cough	0	0.00	12	66.67	4	22.22	2	11.11	0	0.00	0	0.00
Interference of cough with daily activities	1	5.56	15	83.33	1	5.56	1	5.56	0	0.00	0	0.00
Severity of skin dryness	0	0.00	7	38.89	4	22.22	4	22.22	1	5.56	2	11.11
Severity of acne	0	0.00	13	72.22	3	16.67	2	11.11	0	0.00	0	0.00
Severity of itching	0	0.00	12	66.67	4	22.22	2	11.11	0	0.00	0	0.00
Presence of hives	0	0.00	1	5.56	--	--	--	--	--	--	17	94.44
Severity of hand-foot syndrome	0	0.00	16	88.89	1	5.56	1	5.56	0	0.00	0	0.00
Frequency of general pain	0	0.00	12	66.67	3	16.67	3	16.67	0	0.00	0	0.00
Severity of general pain	1	5.56	13	72.22	2	11.11	2	11.11	0	0.00	0	0.00
Interference of general pain with daily activities	1	5.56	16	88.89	0	0.00	1	5.56	0	0.00	0	0.00
Severity of numbness or tingling in the hands and feet	0	0.00	13	72.22	4	22.22	1	5.56	0	0.00	0	0.00
Numbness or tingling in the hands and feet interferes with daily activities	1	5.56	16	88.89	1	5.56	0	0.00	0	0.00	0	0.00
Severity of fatigue	0	0.00	9	50.00	6	33.33	2	11.11	1	5.56	0	0.00
Fatigue interferes with daily activities	1	5.56	12	66.67	2	11.11	2	11.11	0	0.00	1	5.56
Presence of bruising	0	0.00	1	5.56	--	--	--	--	--	--	17	94.44
Other symptoms	0	0.00	13	72.22	1	5.56	3	16.67	1	5.56	0	0.00

It was possible to observe that four symptoms were reported with maximum degree (severity of dry mouth, frequency of diarrhea, severity of skin dryness, interference of fatigue in daily activities). There are seven symptoms with reports of severe degree (frequency of nausea, frequency of diarrhea, severity of shortness of breath, interference of shortness of breath in daily activities, severity of skin dryness, severity of fatigue, other symptoms). The highest percentage of responses is found in the item “none”. In the items “mild” and “moderate”, there are some responses. The questions about the presence of hives (itchy red bumps on the skin)?) and bruising (black and blue marks?) have “yes” or “no” answers, and there was only one positive response for each of these items.

Table 4 presents sample characterization in relation to understandability and adequacy assessment of *PRO-CTCAE^®^ Estudo PROM-TKI Brasil*. None of the respondents marked the alternative “totally disagree” or “disagree”, meaning that participants were able to understand the item and were able to self-assess and choose the alternative that indicated how they felt.

**Table 4:** Sample characterization in relation to understandability and adequacy assessment of PRO-CTCAE^®^ Estudo PROM-TKI Brasil *PRO-CTCAE^®^ Estudo PROM-TKI Brasil* questions

PRO-CTCAE® Estudo PROM-TKI Brasil	I can understand what is written										I can self-assess and choose the alternative that indicates how I am doing									
	Did not answer		Totally disagree		Disagree		Agree		Totally agree		Did not answer		Totally disagree		Disagree		Agree		Totally agree	
	n	%	n	%	n	%	n	%	n	%	n	%	n	%	n	%	n	%	n	%
1	0	0.00	0	0.00	0	0.00	4	22.22	14	77.78	0	0.00	0	0.00	0	0.00	4	22.22	14	77.78
2	0	0.00	0	0.00	0	0.00	3	16.67	15	83.33	1	5.56	0	0.00	0	0.00	3	16.67	14	77.78
3	1	5.56	0	0.00	0	0.00	3	16.67	14	77.78	0	0.00	0	0.00	0	0.00	3	16.67	15	83.33
4	0	0.00	0	0.00	0	0.00	3	16.67	15	83.33	1	5.56	0	0.00	0	0.00	3	16.67	14	77.78
5	0	0.00	0	0.00	0	0.00	3	16.67	15	83.33	1	5.56	0	0.00	0	0.00	3	16.67	14	77.78
6	0	0.00	0	0.00	0	0.00	3	16.67	15	83.33	1	5.56	0	0.00	0	0.00	3	16.67	14	77.78
7	0	0.00	0	0.00	0	0.00	4	22.22	14	77.78	1	5.56	0	0.00	0	0.00	3	16.67	14	77.78
8	0	0.00	0	0.00	0	0.00	3	16.67	15	83.33	1	5.56	0	0.00	0	0.00	3	16.67	14	77.78
9	0	0.00	0	0.00	0	0.00	3	16.67	15	83.33	1	5.56	0	0.00	0	0.00	3	16.67	14	77.78
10	0	0.00	0	0.00	0	0.00	3	16.67	15	83.33	1	5.56	0	0.00	0	0.00	3	16.67	14	77.78
11	0	0.00	0	0.00	0	0.00	3	16.67	15	83.33	1	5.56	0	0.00	0	0.00	3	16.67	14	77.78
12	0	0.00	0	0.00	0	0.00	3	16.67	15	83.33	1	5.56	0	0.00	0	0.00	3	16.67	14	77.78
13	0	0.00	0	0.00	0	0.00	3	16.67	15	83.33	1	5.56	0	0.00	0	0.00	3	16.67	14	77.78
14	0	0.00	0	0.00	0	0.00	3	16.67	15	83.33	1	5.56	0	0.00	0	0.00	3	16.67	14	77.78
15	0	0.00	0	0.00	0	0.00	6	33.33	12	66.67	1	5.56	0	0.00	0	0.00	3	16.67	14	77.78
16	0	0.00	0	0.00	0	0.00	3	16.67	15	83.33	1	5.56	0	0.00	0	0.00	3	16.67	14	77.78
17	0	0.00	0	0.00	0	0.00	3	16.67	15	83.33	1	5.56	0	0.00	0	0.00	3	16.67	14	77.78
18	0	0.00	0	0.00	0	0.00	3	16.67	15	83.33	1	5.56	0	0.00	0	0.00	4	22.22	13	72.22
19	2	11.11	0	0.00	0	0.00	2	11.11	14	77.78	2	11.11	0	0.00	0	0.00	2	11.11	14	77.78
20	2	11.11	0	0.00	0	0.00	3	16.67	13	72.22	3	16.67	0	0.00	0	0.00	4	22.22	11	61.11

In relation to content validity investigation in relation to each question and in relation to understandability and suitability, the results demonstrated that, as a whole, *PRO-CTCAE^®^ Estudo PROM-TKI Brasil* reached the maximum CVI value, i.e., 1.0. Thus, the instrument exceeded the minimum value (≥0.8)^(48)^ and the recommended value (≥0.9) advocated by other authors^(49,50)^.

The maximum value considering understandability and suitability separately was also observed. Finally, for each question considered separately, the maximum CVI was also reached for both suitability and understandability.

## DISCUSSION

*PRO-CTCAE^®^ Estudo PROM-TKI Brasil* was developed based on an integrative review that comprised a set of symptoms confirmed in content validity by experts plus suggestions. During pilot testing, it was found that this version is adequate and understandable from users’ perspective.

With the expansion of the use of TKIs, the need to recognize their specific AEs in order to adopt preventive and treatment measures has become clear. Moreover, from the perspective of quality in oncological care, the need to associate prevalent AEs with appropriate interventions to avoid, resolve or mitigate their harm, achieving safe management, has been outlined. Any new technology, new drugs or new studies bring many benefits, but at the same time, new threats to patient safety. The implementation of tools that provide support for action in routine practice is important in the development of care^(5)^.

AEs occur with varying frequency and intensity in different TKIs. Although it is not the purpose of this article to classify AEs by drug, it is the responsibility of healthcare professionals to differentiate each one for better care planning^(2,3)^. Knowing the frequency, intensity and interference with patients’ quality of life can be a determining factor in planning early management of the most susceptible patients.

PRO-CTCAE™ allows assessing degrees of measurement, such as symptom severity, frequency and its interference with quality of life. Construct, reliability and understandability validity was successful^(53)^.

Using a validated instrument provides additional information to support the care team. PRO-CTCAE™ provides a systematic progression of symptoms, providing clarity and consistency in the recording of care^(54)^.

Chronic illness encourages patients to understand their health status and treatment, enabling them to say something about what they are going through or feeling. The crucial point for the care team is to decide which AEs will be investigated by PRO-CTCAE™, whether electronically or in print. To do this, it is necessary to survey AEs related to indicated treatment^(55)^. It is therefore necessary to think of alternatives that make its use a useful tool not only in clinical research, but also in any care planning.

In order to select items, it is necessary to have prior knowledge of what is being treated. This was the action of phase 1 of this study, when searching the literature for all AEs of a given drug class in order to create an easy-to-use PROM. The integrative literature review provided the theoretical basis for constructing an initial version, aimed at therapeutic intervention with TKI drugs.

According to Kluetz^(55)^, the choice of items should be based on their relevance, but with the aim of reducing redundancy in the questions. Making the questionnaire more flexible makes the tool more easily adaptable to different diseases and treatments^(55)^.

In a study of lung cancer patients divided into two groups (group with osimirtinib and control group with conventional chemotherapy), 28 items from PRO-CTCAE™ were chosen for comparison of AEs. It was found that diarrhea was the most common AE in the group with osimirtinib, and nausea, vomiting and decreased appetite were greater in the conventional chemotherapy group^(45)^.

Two studies used a specific method to select PRO-CTCAE™ items for cancer patients. Selection was based on information from the FDA, EMA, randomized controlled trials, medical record audits, and patient interviews. The questionnaires were well accepted and considered useful by patients^(46,47)^.

In another study, 28 items from PRO-CTCAE™ were selected and administered to patients from seven hospitals, with varying cancer diagnoses and different educational levels, with the aim of verifying the PRO-CTCAE™ equivalence and acceptability in tablet, voice and paper forms. The results demonstrated that answering the questionnaire was not laborious even for individuals with some degree of functional limitation^(48)^.

Piloting is an important phase of studies that have intervention in their designs. In a study that aimed to perform construct validity and reliability testing of PRO-CTCAE™ items, it was suggested that the choice of items for use depends on consultation with specialists, patient suggestions and literature review^(56)^.

The professionals who deal with these individuals are the people who have in their practice the constant reporting and testimony of AEs that occurred with medications, and were, therefore, considered qualified to give an opinion on the relevance or not of the items chosen through literature review^(56)^.

The literature shows that testing PROM-type instruments is essential to validate translation and cultural understanding in the user population^(57)^. The results of this investigation were promising in this sense, strengthening the subsequent phase, which will be to validate the effectiveness.

Concerning the symptoms found, the largest percentage of symptoms were not present in patients, which may mean that they are not symptoms that patients present or that the management of these symptoms is effective and therefore they are not perceived. The hives symptom, which was included in the 2^nd^ round of the Delphi technique at the suggestion of two judges, was identified by 17 patients in pilot testing, showing that it is a symptom that deserves to be included in the instrument, as well as the bruising symptom, which reached the same number of notes. No suggestions of symptoms for inclusion were obtained in pilot testing.

As for participants’ educational level, 88.88% had completed high school or higher education, which may suggest an ease in the self-assessment process and, consequently, a greater ability to communicate with the care team regarding decision-making about their care. These results may suggest that further studies should be carried out to verify the relationship between the population’s literacy level and their understanding and participation in their care^(58)^.

With regard to the understanding of questions, CVI = 1 (Table 5) proves that the questions are written in a way that facilitates patients’ understanding. It is the responsibility of healthcare professionals to promote content that is easy to understand for lay populations^(58)^.

The fact that the National Cancer Institute already has a Portuguese version of PRO-CTCAE™ makes it easier to use in Brazil, and it is up to Brazilian professionals to develop a creative use aimed at our population. This was achieved in the study phases reported in this article.

For cancer patients receiving oral antineoplastic therapy, the concept of self-management gains a new perspective. Patient engagement in their self-care and in the daily routine imposed by a therapeutic plan, incorporating new learning that includes knowledge of possible AEs, their gradations and specific care, should qualify them for self-management. It is evident that patients’ participatory and proactive behavior positively interferes in the survival of chronic diseases. The opposite also happens. Subject to this condition, even if professionals come together to offer the best care, if patients present passive or non-participatory behaviors, treatment evolution progresses with worse outcomes^(59-61)^.

The profile of a proactive patient includes excellent adherence to medication (antineoplastics and others), adoption of preventive behaviors, from oral hygiene to the use of products or any other behavior change. Complementarily, but no less important, proactivity must also be expressed in emotional self-management, in the perception of new emotions that join with past and often conflicting feelings^(59,60)^.

The multidisciplinary team must have the skills to assess and diagnose patients’ deficits, risk situations, multidimensional strengths and weaknesses, including the family in this global assessment. The family can facilitate the process or, on the contrary, can be a reason that is hindering patients’ adaptation to this new phase of treatment. Including *PRO-CTCAE^®^ Estudo PROM-TKI Brasil* in consultations, conversations, guidance and creating bonds are crucial actions for the harmony of the patient/family/nurse relationship^(61)^.

Undoubtedly, the importance of quality teamwork must be emphasized, with emphasis on effective interprofessional communication in daily work and the creation of strategies that promote the discussion of cases in order to generate shared decisions^(62)^. In this regard, patients and family can be incorporated into this team.

### Study limitations

What may have compromised the results was all experts’ non-adherence in the Delphi rounds, although the responses obtained were statistically sufficient for the continuity and veracity of the study. Experts’ adherence is one of the challenges of e-Delphi, as it may lead to non-response due to forgetting the established deadline or not having read the messages received within the established deadline for sending the response. Another important limitation of this study lies in the development of other oral TKI medications that may present signs and symptoms not covered in the stages of this study, configuring the future need for revision of the proposed instrument.

### Contributions to nursing, health or public policy

*PRO-CTCAE^®^ Estudo PROM-TKI Brasil* is the first PROM specific to AEs caused by TKIs. Encouraging the use of PROM in oncology care practice is desirable, and *PRO-CTCAE^®^ Estudo PROM-TKI Brasil* has shown that it can be understood by patients and used in clinical practice so that healthcare professionals have a correct understanding of symptoms and their intensity from the perspective of patients themselves.

*PRO-CTCAE^®^ Estudo PROM-TKI Brasil* may promote excellence in care by requiring rapid and efficient management strategies for AE treatment as well as ensuring the retention of competent experts for continuous assessment and monitoring. The instrument may be a useful tool for patient and family involvement in care and facilitating patient/family recognition of common AEs. It is noteworthy that the inclusion of open-ended questions in the instrument allows for additional reporting of symptoms that may be exclusive to a specific new TKI. The beneficial effects of *PRO-CTCAE^®^ Estudo PROM-TKI Brasil* in clinical care practice need to be assessed in a clinical study designed for this purpose.

## CONCLUSIONS

*PRO-CTCAE^®^ Estudo PROM-TKI Brasil* can be used in healthcare practice and research, because it was subjected to a rigorous construction process, validity by expert judges and assessment of understandability and suitability by patients using TKI medications.
