# Supplementary material for: Outcome measures reported by cancer patients treated with tyrosine kinase inhibitors: a methodological study
Source: Rev Bras Enferm. 2025 Mar 31;78(2):e20240018. doi: 10.1590/0034-7167-2024-0018 (PMC11961046; doi:10.1590/0034-7167-2024-0018)
Supplement: Translation

# Translation: Medidas de resultados relatados pelos pacientes com câncer tratados com inibidores de tirosina-quinase: estudo metodológico

**DOI:** 10.1590/0034-7167-2024-0018pt

**Authors:** Ana Maria Teixeira Pires, Julie Ann Ponto, Eliana Cavalari Teraoka, Fabio Rodrigues Kerbauy, Edvane Birelo Lopes De Domenico

**Affiliations:** I Universidade Federal de São Paulo. São Paulo, São Paulo, Brasil; II Winona State University. Rochester, Minessota, Estados Unidos da América

**Keywords:** Medidas de Resultados Relatados pelo Paciente, Proteínas Tirosina Quinases, Sinais e Sintomas, Assistência Centrada no Paciente, Estudo de Validação.

## Abstract

**Objetivos:**

validar o conteúdo de um instrumento de medidas de resultados com juízes especialistas e avaliar a compreensibilidade e adequabilidade deste instrumento validado com pacientes com câncer sob terapia com inibidores de tirosina-quinase (TKI).

**Métodos:**

estudo metodológico, que incluiu o desenvolvimento de um instrumento através de revisão integrativa, validação de conteúdo, com emprego de técnica Delphi com juízes especialistas, e teste piloto, para verificar compreensibilidade e adequabilidade dos usuários ao instrumento.

**Resultados:**

a revisão de literatura permitiu construir o PRO-CTCAE^®^ Estudo PROM-TKI Brasil, com 16 itens, posteriormente submetido à validação pela técnica Delphi. Após a segunda rodada, o instrumento final foi composto por 20 itens, com Índice de Validade de Conteúdo (IVC) de 0,878. O teste piloto evidenciou que o instrumento é compreensível e adequado à população-alvo (IVC de 1,0).

**Conclusões:**

o PRO-CTCAE^®^ Estudo PROM-TKI Brasil obteve evidências de validação para ser utilizado na vigência de terapia com TKI.

## INTRODUÇÃO

O câncer continua sendo um desafio mundial, com cerca de 19,3 milhões de novos casos (18,1 milhões, excluindo-se o câncer de pele não melanoma) e quase 10,0 milhões de mortes (9,9 milhões, excluindo-se o câncer de pele não melanoma), de acordo com dados de 2020^(1)^.

Entretanto, o combate ao câncer por meio de terapia medicamentosa ganhou fortes aliados, como as terapias-alvo, que têm, em receptores e marcadores celulares específicos, suas principais vias de combate. O uso isolado desses novos fármacos ou combinados com quimioterápicos citotóxicos tem se traduzido em melhores prognósticos dos pacientes em diferentes tipos de câncer^(2)^.

Entre os avanços, os estudos da biologia do câncer permitiram conhecer estruturas moleculares e vias de sinalização de receptores tirosina-quinase, que geraram vários medicamentos chamados então de inibidores das proteínas tirosina-quinase (TKI)^(3)^. Esses fármacos são geralmente bem tolerados, costumam ter um perfil de toxicidade previsível e menos graves do que a quimioterapia citotóxica, o que não significa que não seja necessário manejar seus efeitos e evitar quadros agravados que indiquem comprometimento importante da funcionalidade ou necessidade de intervenção emergencial (normalmente classificados como graus 3 e 4, respectivamente), ocasiões que ensejam a interrupção do tratamento de forma inevitável e, com isso, pior chance de bom desfecho^(4)^.

Reconhecer os efeitos adversos (EAs) é o primeiro passo para adotar condutas preventivas e terapêuticas. Dentro da premissa de prover qualidade e segurança do paciente, há a implantação de ferramentas que oferecem subsídios para atuação na prática clínica, caracterizando os EAs e identificando intervenções capazes de produzir ações seguras e efetivas. Entretanto, invariavelmente, o profissional de saúde é quem faz uma estimativa da gravidade do EA relatado pelo paciente, e, nem sempre, esta é fiel à realidade^(5)^.

Certamente, a capacidade de automonitorar as condições relacionadas aos sinais e sintomas precisa ser desenvolvida nos pacientes, e os instrumentos facilitadores devem ser adequados à cultura de cada país. As ferramentas do tipo *Patient Reported Outcome Measures* (PROMs) permitem ao paciente refletir sobre sua condição e definir o que é importante na sua perspectiva, encorajando-o a discutir seus problemas e melhorar a comunicação com os profissionais de saúde^(6)^.

A escala PRO-CTCAE™, *Patient Reported Outcome* (PRO) - *Common Terminology Criteria for Adverse Events* (CTCAE), é um modelo de ferramenta PROM do *National Cancer Institute*, Estados Unidos da América, composta por 78 itens, englobando todos os sintomas possíveis para o espectro terapêutico oncológico^(6)^. Sua criação baseou-se na leitura de todos os itens do CTCAE que poderiam ser reportados pelos pacientes. Então, descritores específicos para cada item foram selecionados com base em outros questionários utilizados na oncologia, que incluíam graus de medida, como gravidade do sintoma, frequência e a sua interferência na qualidade de vida, com escore de 0 a 4 para escala Likert de 5 pontos (nenhum, leve, moderado, grave e muito grave), incluindo itens como presença/ausência, frequência, severidade e interferência com as atividades diárias.

O paciente é convidado a responder, tendo como orientação “marque ou faça um X na única caixa que melhor descreve suas experiências nos últimos 7 dias”^(6)^. A partir daí, somaram-se os sintomas com uma, duas ou três destas perguntas. As validações de construto, confiabilidade e compreensibilidade foram exitosas^(6)^.

A PRO-CTCAE™ permite não só avaliar os sinais e sintomas dos pacientes, mas fazê-los participar ativamente do seu cuidado em um momento em que a experiência do paciente tem sido tão valorizada e estudada^(6)^.

O profissional pode escolher previamente ou durante a terapêutica quais das toxicidades o paciente está suscetível e buscar correspondência no PRO-CTCAE™, devendo também escolher a periodicidade e o tempo de início da coleta desta informação. O desenvolvimento e a validação do PRO-CTCAE™ foram consistentes e bem estabelecidos pela *US Food and Drug Administration* (FDA) e *European Medicines Agency* (EMA)^(6)^. O processo incluiu pacientes com câncer, profissionais com *expertise* em oncologia, instrumentos de desenvolvimento, pesquisa clínica e aspectos regulatórios.

Testes de validação, confiabilidade e psicométricos em inglês e espanhol foram realizados. Os dados podem ser obtidos através da *web*, sistema interativo de resposta por voz e papéis impressos, oferecendo flexibilidade para escolha que melhor for para a população estudada. O sistema informatizado pode incluir alertas e alarmes para sintomas severos^(6)^, direcionando-o para a equipe assistencial, quer seja através de telemedicina ou até a ida presencial ao setor de origem ou ao pronto-socorro.

Utilizar o PRO-CTCAE™ na íntegra, na prática assistencial, não é factível pelo número de sintomas que reúne. Assim, considerando a necessidade de monitorização efetiva e o cuidado centrado nos pacientes com câncer em uso de fármacos TKI, desenhou-se estudo do tipo intervenção complexa, segundo proposta do *UK Medical Research Council* (MRC)^(7)^. As etapas presentes neste artigo são todas que antecedem o ensaio clínico propriamente dito. Como primeira etapa, realizou-se revisão de literatura e, na sequência, as etapas de validação de conteúdo e pilotagem. As perguntas norteadoras foram: quais EAs os medicamentos tipo TKI causam nos pacientes? Como os pacientes avaliam a compreensibilidade e a adequabilidade de um instrumento do tipo PROM específico para sintomas causados por fármacos TKI?

## OBJETIVOS

Validar o conteúdo dos itens do PRO-CTCAE^®^ Estudo PROM-TKI Brasil com juízes especialistas e avaliar a compreensibilidade e adequabilidade deste instrumento validado com pacientes com câncer sob terapia com TKI.

## MÉTODOS

### Aspectos éticos

O estudo foi aprovado pelo Comitê de Ética em Pesquisa da Universidade Federal de São Paulo (UNIFESP) e do Hospital Alemão Oswaldo Cruz. O estudo está registrado no Registro Brasileiro de Ensaios Clínicos (https://ensaiosclinicos.gov.br/).

### Desenho, período e local de estudo

Trata-se de estudo metodológico, desenvolvido em quatro etapas: revisão integrativa; desenvolvimento do instrumento a partir da revisão; validação de conteúdo dos itens do novo instrumento por meio da técnica Delphi com especialistas; e teste piloto com o público-alvo para avaliação de adequabilidade e compreensibilidade do instrumento (etapas concluídas e descritas neste artigo)^(8)^. Cada etapa de estudo foi baseada em procedimentos metodológicos específicos, e, para assegurar a qualidade da descrição das etapas da pesquisa, foi utilizado o *checklist Standards for Quality Improvement Reporting Excellence* (SQUIRE) 2.0^(9)^.

O estudo iniciou-se em março de 2021, na sua primeira fase de execução, e estendeu-se até o teste piloto, finalizado em setembro de 2022. O teste piloto foi multicêntrico, do qual participaram dois hospitais gerais, um privado e um público, da cidade de São Paulo, São Paulo, Brasil, e que possuem centros de atenção oncológica, incluindo unidades ambulatoriais.

### Protocolos dos estudos

Os protocolos de estudo concernentes a cada etapa estão detalhados na sequência da ocorrência.

### Etapa 1: Revisão integrativa

A revisão integrativa da literatura proporcionou a busca, avaliação crítica e a síntese das evidências disponíveis sobre o tema investigado^(10)^. Para nortear a busca por artigos científicos, utilizou-se a estratégia SPIDER, por ser mais adequada para estudos de natureza mista e/ou qualitativa exclusivamente e por ajustar-se aos objetivos da investigação^(11)^. Os elementos que compuseram o acrônimo SPIDER encontram-se no Quadro 1 e permitiram a formulação da pergunta de estudo: quais EAs dos TKI (PI e E) em pacientes com câncer (S) estão relatados na literatura (D e R)?

**Quadro 1:** Descritores selecionados pela técnica SPIDER

SPIDER	Descritores
*Sample*	*“oncology patients”*
*Phenomenon of Interest*	*“protein kinase inhibitor” OR* *“tyrosine kinase inhibitor” AND “adverse effects” OR “adverse drug reaction”*
*Design*	*“meta-analysis” OR “systematic review” OR “review”*
*Evaluation*	*“adverse effects” OR “adverse drug reaction” AND “tyrosine kinase inhibitor”*
*Research type*	*“quantitative” OR “qualitative”, “mixed methods”*

Os critérios de inclusão adotados para a seleção dos artigos foram apenas revisões de literatura, quer fossem integrativas, sistemáticas com ou sem meta-análise, disponíveis na íntegra, nos idiomas português, espanhol e inglês, no período de 2018 a fevereiro de 2021, nas bases de dados *National Library of Medicine* (PubMed), *Web of Science* e Embase.

Foram selecionados 140 artigos das três bases de dados seguindo o critério *Preferred Reporting Items for Systematic Reviews and Meta-Analyses* (PRISMA) (Figura 1). Estes artigos foram transferidos para o aplicativo Rayyan^(12)^. Este aplicativo permite analisar cada título, identificá-lo como texto incluso ou não, detalhar o motivo da exclusão e fazer pequenas anotações. Essas ações facilitaram a contagem final e registro de todas as fases do método utilizado.

**Figura 1:** Fluxograma do modelo PRISMA sobre seleção dos artigos, 2021

Após a leitura dos títulos, 107 artigos foram excluídos, e os principais motivos foram tratar-se de outros medicamentos, patologias não oncológicas e outros métodos de pesquisa. Na leitura dos 33 resumos, foram retirados oito por não responderem às perguntas de estudo e um por duplicidade. Os 24 artigos restantes que atendiam aos critérios de inclusão compuseram a amostra final. A leitura do artigo foi feita na íntegra para avaliar o delineamento do estudo e procurar os dados de interesse da pesquisa, selecionando-se as frases que descreviam os EAs dos TKI^(13-36)^. Todos os EAs foram listados e organizados em grupos para posterior busca do item no instrumento original PRO-CTCAE™, exceto aqueles que só apareceram em um artigo (visão embaçada, lesão na córnea, blefarite, olho seco, alteração da voz).

### Etapa 2 - Desenvolvimento do PRO-CTCAE^®^ Estudo PROM-TKI Brasil

Para cada EA encontrado mais de uma vez na revisão integrativa, foi buscado item análogo no instrumento original PRO-CTCAE™. Foram escolhidos, portanto, 16 itens do documento acrescido de uma questão aberta, também constante do original, para a criação da versão PRO-CTCAE^®^ Estudo PROM-TKI Brasil.

### Etapa 3 - Validação de conteúdo do PRO-CTCAE^®^ Estudo PROM-TKI Brasil

A validade é um fator crítico na seleção e aplicação de um instrumento na prática e na pesquisa, e significa até que ponto o instrumento mede o que pretende medir^(37,38)^. Para testar o estado de saúde do paciente, por exemplo, o *Food and Drug Administration* (FDA, USA) fornece orientação para avaliar e revisar os instrumentos do tipo PRO usados para medir o tratamento, benefício ou risco associado a produtos médicos, com base em sua estrutura conceitual e validade de conteúdo. O FDA incentiva os desenvolvedores de instrumentos a fornecer evidências do conteúdo do instrumento usando revisão de literatura e opiniões dos especialistas e pacientes-alvo^(37,38)^.

A validação de conteúdo contempla a participação de especialistas no assunto para avaliar a relevância dos conteúdos e a extensão com que cada item do instrumento comprova o fenômeno de interesse e a dimensão de cada um no conjunto do que se pretende avaliar^(38)^.

A técnica Delphi permite reunir opiniões de especialistas, sem restrições às áreas geográficas, sobre temáticas variadas e complexas^(39)^. Na prática, os especialistas respondem a um questionário estruturado, de forma anônima, por meio de rodadas. Os dados da primeira rodada são analisados pelo pesquisador, e calcula-se quantitativamente a concordância. Na sequência, são enviados o resumo das modificações, a análise quantitativa da concordância e o documento reformulado para os mesmos especialistas, conformando a 2ª rodada e assim por diante. As rodadas são finalizadas a partir do consenso entre os especialistas, também chamados de juízes ou *experts* ^(39)^.

### Local, amostra, critérios de inclusão e exclusão

Foram convidados médicos oncologistas e enfermeiros especialistas em oncologia que integram ambas as instituições participantes do estudo.

Para a inclusão, elegeram-se médicos oncologistas e enfermeiros que somaram 4 pontos ou mais na classificação adaptada de Fehring, que consiste na pontuação do especialista de acordo com a sua atuação profissional, titulação e produção de conhecimentos na área de conhecimento investigada^(40)^. A área de conhecimento dos juízes considerou a experiência com pacientes sob terapia com TKI tanto na área de pesquisa quanto no cuidado diário. Os médicos deviam ser prescritores e responsáveis pelo cuidado a esses pacientes, e os enfermeiros deviam atuar na prática com esta terapia. O critério de exclusão consistiu no preenchimento parcial de um dos instrumentos de coleta de dados.

### Procedimentos para a coleta de dados

A seleção de participantes se deu por mensagem por correio eletrônico individualizado, esclarecimento de dúvidas, aceite de participação, assinatura do Termo de Consentimento Livre e Esclarecido (TCLE), segundo as recomendações da Resolução nº 466/12 do Conselho Nacional de Saúde (CNS), e, na sequência, os dados foram coletados.

Foram utilizados dois instrumentos estruturados: o primeiro com identificação pessoal (sexo e idade) e atividade profissional, e o segundo, específico para a validação de conteúdo. Não houve identificação nominal dos respondentes em documento, sendo codificados com números sequenciais. O PRO-CTCAE^®^ Estudo PROM-TKI Brasil foi enviado e esclarecido com relação à sua construção. Cada item do instrumento (sintoma) teve sua avaliação baseada em critérios julgados oportunos para a intencionalidade da investigação, como objetividade, simplicidade, clareza, relevância e precisão^(38)^. Para cada item, o juiz assinalou uma das alternativas da escala Likert: 1: discordo totalmente; 2: discordo; 3: concordo; 4: concordo totalmente. Ao final de cada item, havia a possibilidade de inserir alguma observação e, no final de todos os itens, havia a pergunta “Existe algum outro sintoma que o(a) Sr(a) acha necessário ser incluído? Se sim, qual?”, que gerou a possibilidade de sugerir outros sintomas não apresentados e que julgavam serem importantes no manejo clínico do paciente sob terapia oral com TKI.

Para a análise do processo de cada rodada da Delphi, foi calculado o Índice de Validade de Conteúdo (IVC). A concordância mínima desejável foi de 0,80^(41)^.

### Etapa 4 - Teste piloto

O instrumento construído após a revisão de literatura e a validação com os juízes especialistas foi utilizado para o teste piloto para avaliar a compreensibilidade e adequabilidade do instrumento após validação de conteúdo, agora pelos usuários finais, pacientes em uso oral de fármacos TKI. Para avaliar os objetivos propostos, uma amostra pequena de participantes poderá gerar as respostas desejadas ou utilizar a porcentagem de 10% da amostra do estudo da próxima fase^(42)^. O tamanho amostral da próxima fase será por conveniência, com base nas recomendações que consideram que, para um nível de 5% de significância, é desejável incluir de cinco a dez indivíduos por item do questionário^(43)^. Como o questionário contém 20 itens, a amostra será de 100 a 200 pacientes, sendo, portanto, neste piloto, o indicado de dez a 20 pacientes.

O teste piloto foi desenhado para avaliar as seguintes hipóteses:

H1: PRO-CTCAE^®^ Estudo PROM-TKI Brasil é de fácil compreensão e fácil preenchimento;

H2: PRO-CTCAE^®^ Estudo PROM-TKI Brasil contempla os sintomas apresentados pelos pacientes.

### Local, amostra, critérios de inclusão e exclusão

Foram convidados pacientes das duas instituições, aleatoriamente, nos dias de atendimentos médicos ambulatoriais, constituindo uma amostra por conveniência.

Os critérios de inclusão consistiram de pacientes maiores de 18 anos, com diagnóstico de tumor de rim, pulmão ou leucemia mieloide crônica que realizam tratamento com qualquer TKI há mais de dois meses e com funcionalidade clínica avaliada pela escala *Eastern Cooperative Oncologic Group* (ECOG) entre 0 e 2. Os critérios de exclusão foram possuir múltiplas comorbidades (≥ 3) e/ou preenchimento parcial de um dos instrumentos de coleta de dados.

### Procedimentos para a coleta de dados

Após a seleção dos participantes, foi feito um contato pessoal no dia da consulta médica utilizando um roteiro pré-estabelecido, convidando-o para participar, e, neste momento, as informações foram fornecidas e, após a assinatura do TCLE, os instrumentos foram aplicados. O instrumento de caracterização do participante constou de dados sociodemográficos e clínicos (idade, sexo, diagnóstico da doença oncológica, doença pré-existente e medicamentos em uso).

O participante foi orientado a responder ao PRO-CTCAE^®^ Estudo PROM-TKI Brasil validado na etapa anterior, apresentado na forma impressa, após assinatura do TCLE. Para cada item de sintoma constante no instrumento, havia duas afirmativas: “consigo compreender o que está escrito” e “consigo me autoavaliar e escolher a alternativa que indica como estou”. As alternativas para cada afirmativa estavam em escala tipo Likert com 4 pontos: 1: discordo totalmente; 2: discordo; 3: concordo; 4: concordo totalmente. Na sequência, questionou-se sobre o tamanho e tipo de letra e o formato da apresentação em folheto, acompanhado pelas alternativas acima. Por último, deixou-se um espaço para a escrita de sugestões.

Os dados sociodemográficos foram avaliados de forma descritiva, por meio de números absolutos e percentuais, e para as respostas em escala tipo Likert, foi calculado o IVC, que indica a porcentagem de concordância entre os respondentes. A concordância mínima desejável foi de 0,80^(41)^. Utilizou-se o *software* SPSS *Statistics*, versão 28.0 (IBM Corp., Armonk, NY, EUA). A base teórica utilizada para a análise de dados apresentada neste relatório está descrita de forma pormenorizada por Field^(44)^.

## RESULTADOS

Na etapa de revisão de literatura, selecionaram-se 24 artigos e, nestes, foram encontrados 58 EAs. Posteriormente, optou-se por organizar os EAs em oito grupos. Os agrupamentos foram denominados como gastrointestinal, dermatológico, cardíaco, renal, pulmonar, mielotoxicidade, fadiga e outros (efeitos que não se encaixavam nos itens anteriores).

Dos EAs gastrointestinais, náusea, vômito, perda de apetite, diarreia, constipação e mucosite foram sintomas encontrados no PRO-CTCAE™, sendo a mucosite relacionada com os itens “boca seca” e “feridas na boca ou garganta”. Perda de peso foi um EA encontrado, mas como é um sinal que necessita de medição, não é respondido subjetivamente e, portanto, não tem análogo no PRO-CTCAE™.

Com relação ao sistema tegumentar, os efeitos encontrados foram *rash*, paroníquia, prurido, síndrome mão-pé e xerose. No PRO-CTCAE™ encontraram-se pele seca, coceira, síndrome mão-pé, que foram incluídos. Com relação à paroníquia, o PRO-CTCAE™ contém três itens relacionados à unha que são conceitualmente diferentes (se houve perda de alguma unha, presença de protuberância na unha e alteração da cor da unha) e, portanto, não foram considerados.

A hepatotoxicidade, alteração da função renal ou cardíaca são sinais obtidos através de exames laboratoriais que não são percebidos pelos pacientes, portanto, não têm análogos no PRO-CTCAE™. A hipertensão, por exemplo, necessita de medição, assim como é necessário eletrocardiograma para observar alterações cardíacas. Entretanto, a falta de ar pode indicar uma alteração pulmonar, sendo, assim, incluída no instrumento.

Somente um artigo citou edema como EA, mas sem especificação. No PRO-CTCAE™ há três itens sobre edema em locais específicos (inchaço de braços e pernas, inchaço abdominal e inchaço das mamas) que não foram considerados^(13-36)^, pois a citação do artigo não especifica o local do edema, contrário do que se encontra no PRO-CTCAE™. Incluir os três itens sobre inchaço aumentaria o número de itens para uma quantidade que poderia não ser aceitável. Favorecendo essa iniciativa, na segunda rodada, quando foi incluído inchaço abdominal (por sugestão de um juiz na primeira rodada), nenhum dos três juízes aprovou, e o item inchaço de membros superiores e inferiores foi aceito por somente um juiz, com o IVC de 0,33, que invalida sua inclusão.

Quanto à mielotoxicidade, por ser um efeito verificado através de exames laboratoriais, não tem análogo no PRO-CTCAE™. Febre poderia ser um sintoma percebido, mas é principalmente definido através da medição da temperatura, não tendo análogo no PRO-CTCAE™. Fadiga foi um EA encontrado na literatura e está presente no PRO-CTCAE™, tendo sido incluído^(13-36)^.

Algumas alterações metabólicas, como hiperglicemia, hipercolesterolemia, hipotireoidismo e hipocalcemia, foram encontradas na literatura, mas não são sintomas percebidos pelo paciente, não tendo análogo no PRO-CTCAE™.

Com relação à alteração ocular, somente um artigo detalhou sintomas como olho seco, conjuntivite, catarata e lesão na córnea, não sendo incluídos pelo critério de exclusão estabelecido^(13-36)^.

Cada EA encontrado nos mesmos termos no PRO-CTCAE™ foi transferido para um novo documento, conformando 16 itens. Como a última pergunta (17ª) se referia a qualquer sintoma que o paciente poderia sentir, o instrumento seria capaz de estimular o paciente a lembrar de algum outro sintoma não contemplado e responder sobre este, pois as últimas perguntas consistiam de: você tem algum outro sintoma que deseja relatar? Qual? Nos últimos sete dias, qual foi a gravidade desse sintoma na pior situação? O novo documento, a partir de então, foi denominado PRO-CTCAE^®^ Estudo PROM-TKI Brasil.

### Validação de conteúdo do PRO-CTCAE^®^ Estudo PROM-TKI Brasil

A realização da etapa 3 deste estudo (validação do instrumento através de consenso de juízes especialistas) adicionou veracidade na escolha dos itens, visto que, além de utilizar dados de uma revisão de literatura, foram considerados o conhecimento e a prática dos juízes. O primeiro critério para a escolha dos juízes foi ser profissional de qualquer uma das duas instituições do estudo. O segundo critério seguiu a classificação adaptada de Fehring, o que culminou em um grupo experiente de juízes.

No processo de validação, o PRO-CTCAE^®^ Estudo PROM-TKI Brasil foi enviado para 15 juízes (13 médicos oncologistas e duas enfermeiras), individualmente, por correio eletrônico, junto com uma explicação sobre a pesquisa, sua importância e seus objetivos, solicitando resposta e estabelecendo um prazo de 15 dias. Após sete dias, foi encaminhado outro e-mail como lembrete para cada juiz. Após o tempo decorrido, foi enviado novo e-mail aceitando ainda um prazo de mais sete dias. Na primeira rodada, foram recebidos seis TCLEs preenchidos e seis questionários respondidos, correspondendo à amostra da primeira rodada. Os juízes compunham cinco médicos oncologistas (um doutor, dois mestres e dois especialistas com mais de dez anos de atividade na área) e uma enfermeira (mestre com mais de dez anos de atividade na área).

Na primeira rodada, obteve-se o IVC de 0,878, mostrando que a escolha dos itens poderia ser mantida. Através de uma pergunta aberta sobre sugestões de inclusões, os juízes respondentes sugeriram cinco sintomas, que foram incluídos para serem submetidos à validação na segunda rodada. A técnica Delphi permitiu, portanto, através das suas rodadas, melhorar o documento baseado na *expertise* dos juízes. Feito isso, os dados foram analisados, e os três itens que conseguiram o IVC de 0,67 (apesar de menor que 0,8) foram incluídos no questionário, que ficou com um total de 20 perguntas, número razoável para a aceitação dos pacientes^(45-48)^.

A Tabela 1 apresenta os valores do IVC para cada item calculado constante no instrumento inicial PRO-CTCAE^®^ Estudo PROM-TKI Brasil^(49)^.

**Tabela 1:** Índice de Validade de Conteúdo de acordo com a questão e de acordo com o tipo de equivalência para o PRO-CTCAE^®^ Estudo PROM-TKI Brasil

Item	Critério					Média por questão
	Objetividade	Simplicidade	Clareza	Relevância	Precisão	
Gravidade da boca seca^*^	0,83	0,67	0,67	0,83	0,83	0,77
Gravidade de feridas na boca	1,00	0,83	0,83	1,00	0,83	0,90
Interferência das feridas na boca nas atividades diárias	1,00	1,00	0,83	1,00	0,83	0,93
Gravidade da diminuição do apetite	1,00	0,83	0,83	1,00	0,83	0,90
Interferência da diminuição do apetite nas atividades diárias^*^	0,67	0,67	0,33	0,83	0,50	0,60
Frequência da náusea	1,00	1,00	1,00	1,00	1,00	1,00
Gravidade da náusea^*^	0,83	0,83	0,67	1,00	1,00	0,87
Frequência do vômito^*^	0,83	0,83	0,67	1,00	1,00	0,87
Gravidade do vômito^*^	0,83	0,67	0,67	0,83	0,833	0,77
Gravidade da constipação^*^	1,00	0,83	0,667	1,00	1	0,90
Frequência da diarreia	0,83	0,83	0,83	0,83	0,83	0,83
Gravidade da falta de ar	1,00	0,83	0,833	1,00	1,00	0,93
Interferência da falta de ar nas atividades diárias^*^	1,00	0,83	0,50	1,00	0,83	0,83
Gravidade da tosse^*^	0,83	0,67	0,67	0,83	0,83	0,77
Interferência da tosse nas atividades diárias^*^	0,83	0,83	0,67	0,83	0,83	0,80
Gravidade da secura da pele	1,00	0,83	0,83	0,83	1,00	0,90
Gravidade da coceira	1,00	0,83	0,83	1,00	1,00	0,93
Gravidade da síndrome mão-pé^*^	0,83	0,83	0,67	1,00	0,83	0,83
Gravidade da dormência de mãos e pés	1,00	0,83	0,83	1,00	1,00	0,93
Interferência da dormência nas atividades diárias	1,00	0,83	0,83	1,00	1,00	0,93
Frequência da dor	1,00	1,00	1,00	1,00	1,00	1,00
Gravidade da dor	1,00	0,83	0,83	1,00	1,00	0,93
Interferência da dor nas atividades diárias	1,00	0,83	0,83	1,00	1,00	0,93
Gravidade da fadiga	1,00	0,83	0,83	1,00	1,00	0,93
Interferência da fadiga nas atividades diárias	1,00	0,83	0,83	1,00	1,00	0,93
Presença de hematoma	1,00	1,00	1,00	0,83	1,00	0,97
Outros sintomas^*^	0,83	0,67	0,67	1,00	0,83	0,80
Média por critério	0,93	0,83	0,76	0,95	0,91	0,88

Os resultados da Tabela 1 demonstraram que, como um todo, o PRO-CTCAE^®^ Estudo PROM-TKI Brasil alcançou o IVC mínimo (0,878) proposto por Davis^(50)^, porém não atingiu o IVC recomendado (≥ 0,9) sugerido por Lynn^(51)^ e Waltz^(49)^. Em relação a cada critério, observou-se que o critério clareza ficou abaixo de 0,8 e o critério simplicidade ficou entre 0,8 e 0,899, enquanto que os demais critérios tiveram valor maior ou igual a 0,9.

Em relação ao IVC para cada item, os seguintes itens não alcançaram o IVC mínimo de 0,78 proposto por Lynn^(51)^ para amostras de seis a dez juízes (marcados com asterisco*): boca seca: simplicidade e clareza; interferência da perda de apetite nas atividades diárias: objetividade, simplicidade, clareza e precisão; gravidade da náusea: clareza; frequência do vômito: clareza; gravidade do vômito: simplicidade e clareza; gravidade da constipação: clareza; interferência da falta de ar nas atividades diárias: clareza; gravidade da tosse: simplicidade e clareza; interferência da tosse nas atividades diárias: clareza; gravidade da síndrome mão-pé: clareza; outros sintomas: simplicidade e clareza. Apesar de o critério clareza ter atingido o menor índice (0,76), o teste piloto mostrou uma excelente compreensão (IVC = 1) por parte da população-alvo. Os demais itens alcançaram o IVC mínimo em seus respectivos critérios.

Adicionalmente, na questão aberta, os participantes sugeriram alterações nas redações de alguns itens presentes no PRO-CTCAE™ que modificariam a versão disponibilizada em língua portuguesa, e estas não foram acatadas, pois há regras de uso que restringem qualquer modificação^(52)^. Entretanto, também houve sugestões de sinais e sintomas que não constavam no instrumento, tais como insônia, inchaço abdominal, inchaço de membros superiores e inferiores, acne e urticária.

Após a leitura atenta da versão original do PRO-CTCAE™ pelas pesquisadoras, foram encontrados análogos aos sinais e sintomas sugeridos. Assim, foi criada a versão para a segunda rodada do PRO-CTCAE^®^ Estudo PROM-TKI Brasil com o objetivo de avaliar a concordância em relação aos novos itens. Essa versão foi encaminhada para os especialistas juízes, juntamente com as explicações das alterações realizadas e não realizadas, obtendo-se resposta de três participantes. A Tabela 2 mostra os resultados da segunda rodada, com os valores do IVC para cada item.

**Tabela 2:** Índice de Validade de Conteúdo de sintomas avaliados na segunda rodada de acordo com o item para incorporação no PRO-CTCAE^®^ Estudo PROM-TKI Brasil

Item	IVC
Rachadura boca	0,67
Acne	0,67
Urticária	0,67
Insônia	0,33
Inchaço de braços e pernas	0,33
Inchaço abdominal	0,00

Apesar de o IVC não ter apresentado concordância total (0,67), foram incluídos os sintomas “rachadura no canto da boca”, “acne” e “urticária”, correspondentes ao grupo dermatológico, de acordo com a sugestão de literatura especializada que advoga pela concordância da maioria^(51)^. Os sintomas excluídos foram “inchaço de braços e pernas” e “insônia”, por terem sido aceitos por um juiz apenas (IVC: 0,33), e “inchaço abdominal”, por nenhuma aceitação (IVC: 0,0).

A versão final do PRO-CTCAE^®^ Estudo PROM-TKI Brasil se mostrou adequada para os pacientes sob esta modalidade de tratamento.

### Avaliação do instrumento quanto à compreensibilidade e adequabilidade

A população do teste piloto foi constituída por 18 pacientes das instituições participantes do estudo. A maioria (n:12) era do sexo masculino (66,7%); dez eram usuários do Sistema Único de Saúde (55,6%); oito eram usuários da saúde suplementar (44,4%); quatro eram solteiros (22,2%); 12 eram casados (66,7%); dois eram viúvos (11,1%); um era estudante (6,2%); 11 estavam empregados (68,7); quatro estavam aposentados (25,0%) (dois não responderam). Com relação ao grau de instrução, um tem ensino fundamental incompleto (5,6%); um tem ensino fundamental completo (5,6%); oito têm ensino médico completo (44,4%); oito têm ensino superior completo (4,4%). Na caracterização da funcionalidade clínica, 15 pacientes tinham ECOG (PS) = 0 (83,3%), e três tinham *Performance Status* (PS) = 1 (16,7%). Com relação às comorbidades, cinco pacientes (27,8%) tinham comorbidades isoladas ou acumuladas de diabetes, hipertensão arterial sistêmica, hipercolesterolemia e hipotireoidismo.

Quanto ao diagnóstico e tratamento, dez pacientes foram diagnosticados com leucemia mieloide crônica (55,6%); quatro pacientes foram diagnosticados com câncer de rim (22,2%); três pacientes foram diagnosticados com câncer de pulmão (16,7%); e um paciente foi diagnosticado com linfoma de Hodgkin (5,6%). A maioria (n:13) recebia TKI isolado (72,2%), e cinco pacientes recebiam TKI associado a outra medicação antineoplásica (27,8%). Os fármacos TKI mencionados pelos pacientes foram imatinibe, nilotinibe, axitinibe, ibrutinibe, osimertinibe e alectinibe.

A Tabela 3 apresenta as frequências das respostas assinaladas pelos participantes para cada item do PRO-CTCAE^®^ Estudo PROM-TKI Brasil.

**Tabela 3:** Distribuição da amostra em relação às respostas por item constante no PRO-CTCAE^®^ Estudo PROM-TKI Brasil

Questões do PRO-CTCAE^®^ Estudo PROM-TKI Brasil	Não respondeu		Nenhum/ nunca/ nada/não		Leve/raramente/muito pouco		Moderada/às vezes/um pouco		Grave/frequentemente/bastante		Muito grave/ quase constantemente/muito/sim	
	n	%	n	%	n	%	n	%	n	%	n	%
Gravidade da boca seca	0	0,00	10	55,56	5	27,78	2	11,11	0	0,00	1	5,56
Gravidade de feridas na boca	0	0,00	17	94,44	0	0,00	1	5,56	0	0,00	0	0,00
Interferência das feridas na boca nas atividades diárias	2	11,11	16	88,89	0	0,00	0	0,00	0	0,00	0	0,00
Gravidade de rachadura na boca	0	0,00	17	94,44	1	5,56	0	0,00	0	0,00	0	0,00
Gravidade da diminuição do apetite	0	0,00	16	88,89	2	11,11	0	0,00	0	0,00	0	0,00
Interferência da diminuição do apetite nas atividades diárias	1	5,56	17	94,44	0	0,00	0	0,00	0	0,00	0	0,00
Frequência da náusea	0	0,00	13	72,22	1	5,56	3	16,67	1	5,56	0	0,00
Gravidade da náusea	1	5,56	12	66,67	4	22,22	1	5,56	0	0,00	0	0,00
Frequência do vômito	0	0,00	16	88,89	0	0,00	2	11,11	0	0,00	0	0,00
Gravidade do vômito	1	5,56	15	83,33	1	5,56	1	5,56	0	0,00	0	0,00
Gravidade da constipação	0	0,00	12	66,67	4	22,22	2	11,11	0	0,00	0	0,00
Frequência da diarreia	0	0,00	14	77,78	0	0,00	1	5,56	1	5,56	2	11,11
Gravidade da falta de ar	0	0,00	15	83,33	1	5,56	1	5,56	1	5,56	0	0,00
Interferência da falta de ar nas atividades diárias	1	5,56	16	88,89	0	0,00	0	0,00	1	5,56	0	0,00
Gravidade da tosse	0	0,00	12	66,67	4	22,22	2	11,11	0	0,00	0	0,00
Interferência da tosse nas atividades diárias	1	5,56	15	83,33	1	5,56	1	5,56	0	0,00	0	0,00
Gravidade da secura da pele	0	0,00	7	38,89	4	22,22	4	22,22	1	5,56	2	11,11
Gravidade da acne	0	0,00	13	72,22	3	16,67	2	11,11	0	0,00	0	0,00
Gravidade da coceira	0	0,00	12	66,67	4	22,22	2	11,11	0	0,00	0	0,00
Presença de urticária	0	0,00	1	5,56	--	--	--	--	--	--	17	94,44
Gravidade da síndrome mão-pé	0	0,00	16	88,89	1	5,56	1	5,56	0	0,00	0	0,00
Frequência da dor	0	0,00	12	66,67	3	16,67	3	16,67	0	0,00	0	0,00
Gravidade da dor	1	5,56	13	72,22	2	11,11	2	11,11	0	0,00	0	0,00
Interferência da dor nas atividades diárias	1	5,56	16	88,89	0	0,00	1	5,56	0	0,00	0	0,00
Gravidade da dormência de mãos e pés	0	0,00	13	72,22	4	22,22	1	5,56	0	0,00	0	0,00
Interferência da dormência nas atividades diárias	1	5,56	16	88,89	1	5,56	0	0,00	0	0,00	0	0,00
Gravidade da fadiga	0	0,00	9	50,00	6	33,33	2	11,11	1	5,56	0	0,00
Interferência da fadiga nas atividades diárias	1	5,56	12	66,67	2	11,11	2	11,11	0	0,00	1	5,56
Presença de hematoma	0	0,00	1	5,56	--	--	--	--	--	--	17	94,44
Outros sintomas	0	0,00	13	72,22	1	5,56	3	16,67	1	5,56	0	0,00

Foi possível observar que quatro sintomas foram relatados com grau máximo (gravidade da boca seca, frequência da diarreia, gravidade de secura de pele, interferência da fadiga na atividade diária). Há sete sintomas com relatos de grau grave/frequentemente/bastante (frequência da náusea, frequência da diarreia, gravidade de falta de ar, interferência da falta de ar nas atividades diárias, gravidade de secura da pele, gravidade da fadiga, outro sintoma). Porcentagem maior de resposta se encontra no item “nenhum/nunca/nada/não”. Nos itens “leve/raramente/muito pouco” e “moderada/às vezes/um pouco”, encontram-se algumas respostas. As perguntas sobre presença de urticária (caroços vermelhos que coçam na pele?) e hematomas (marcas pretas e roxas?) possuem respostas “sim” ou “não”, e obteve-se somente uma resposta positiva para cada um desses itens.

A Tabela 4 apresenta a caracterização da amostra em relação à avaliação quanto à compreensibilidade e adequabilidade do conteúdo do PRO-CTCAE^®^ Estudo PROM-TKI Brasil. Nenhum dos pesquisados assinalou a alternativa “discordo totalmente” ou “discordo”, significando que os participantes conseguiram compreender o item e conseguiram se autoavaliar e escolher a alternativa que indicava como se sentiam.

**Tabela 4:** Caracterização da amostra em relação à avaliação quanto à compreensibilidade e adequabilidade do PRO-CTCAE^®^ Estudo PROM-TKI Brasil Questões do PRO-CTCAE^®^ Estudo PROM-TKI Brasil

PRO-CTCAE^®^ Estudo PROM-TKI Brasil	Consigo compreender o que está escrito										Consigo me autoavaliar e escolher a alternativa que indica como estou									
	Não respondeu		Discordo totalmente		Discordo		Concordo		Concordo totalmente		Não respondeu		Discordo totalmente		Discordo		Concordo		Concordo totalmente	
	n	%	n	%	n	%	n	%	n	%	n	%	n	%	n	%	n	%	n	%
1	0	0,00	0	0,00	0	0,00	4	22,22	14	77,78	0	0,00	0	0,00	0	0,00	4	22,22	14	77,78
2	0	0,00	0	0,00	0	0,00	3	16,67	15	83,33	1	5,56	0	0,00	0	0,00	3	16,67	14	77,78
3	1	5,56	0	0,00	0	0,00	3	16,67	14	77,78	0	0,00	0	0,00	0	0,00	3	16,67	15	83,33
4	0	0,00	0	0,00	0	0,00	3	16,67	15	83,33	1	5,56	0	0,00	0	0,00	3	16,67	14	77,78
5	0	0,00	0	0,00	0	0,00	3	16,67	15	83,33	1	5,56	0	0,00	0	0,00	3	16,67	14	77,78
6	0	0,00	0	0,00	0	0,00	3	16,67	15	83,33	1	5,56	0	0,00	0	0,00	3	16,67	14	77,78
7	0	0,00	0	0,00	0	0,00	4	22,22	14	77,78	1	5,56	0	0,00	0	0,00	3	16,67	14	77,78
8	0	0,00	0	0,00	0	0,00	3	16,67	15	83,33	1	5,56	0	0,00	0	0,00	3	16,67	14	77,78
9	0	0,00	0	0,00	0	0,00	3	16,67	15	83,33	1	5,56	0	0,00	0	0,00	3	16,67	14	77,78
10	0	0,00	0	0,00	0	0,00	3	16,67	15	83,33	1	5,56	0	0,00	0	0,00	3	16,67	14	77,78
11	0	0,00	0	0,00	0	0,00	3	16,67	15	83,33	1	5,56	0	0,00	0	0,00	3	16,67	14	77,78
12	0	0,00	0	0,00	0	0,00	3	16,67	15	83,33	1	5,56	0	0,00	0	0,00	3	16,67	14	77,78
13	0	0,00	0	0,00	0	0,00	3	16,67	15	83,33	1	5,56	0	0,00	0	0,00	3	16,67	14	77,78
14	0	0,00	0	0,00	0	0,00	3	16,67	15	83,33	1	5,56	0	0,00	0	0,00	3	16,67	14	77,78
15	0	0,00	0	0,00	0	0,00	6	33,33	12	66,67	1	5,56	0	0,00	0	0,00	3	16,67	14	77,78
16	0	0,00	0	0,00	0	0,00	3	16,67	15	83,33	1	5,56	0	0,00	0	0,00	3	16,67	14	77,78
17	0	0,00	0	0,00	0	0,00	3	16,67	15	83,33	1	5,56	0	0,00	0	0,00	3	16,67	14	77,78
18	0	0,00	0	0,00	0	0,00	3	16,67	15	83,33	1	5,56	0	0,00	0	0,00	4	22,22	13	72,22
19	2	11,11	0	0,00	0	0,00	2	11,11	14	77,78	2	11,11	0	0,00	0	0,00	2	11,11	14	77,78
20	2	11,11	0	0,00	0	0,00	3	16,67	13	72,22	3	16,67	0	0,00	0	0,00	4	22,22	11	61,11

Quanto à investigação da validade de conteúdo em relação a cada questão e em relação à compreensibilidade e adequabilidade, os resultados demonstraram que, como um todo, o PRO-CTCAE^®^ Estudo PROM-TKI Brasil alcançou o valor máximo de IVC, ou seja, 1,0. Assim, o instrumento superou o valor mínimo (≥0,8)^(48)^ e o valor recomendado (≥ 0,9) defendidos por outros autores^(49,50)^.

Também foi observado o valor máximo considerando a compreensibilidade e a adequabilidade isoladamente. Por fim, para cada questão considerada de forma isolada, também foi atingido o IVC máximo tanto para adequabilidade quanto para compreensibilidade.

## DISCUSSÃO

O PRO-CTCAE^®^ Estudo PROM-TKI Brasil foi elaborado a partir de uma revisão integrativa que compôs um conjunto de sintomas confirmados na validação de conteúdo pelos especialistas, acrescidos de sugestões. Na pilotagem, verificou-se que esta versão está adequada e é compreensível na perspectiva dos usuários.

Com a ampliação do uso dos TKI, ficou clara a necessidade de reconhecer seus EAs específicos para adoção de condutas preventivas e de tratamento. De forma complementar, na perspectiva da qualidade da assistência oncológica, delineia-se a necessidade de associar os EAs prevalentes às intervenções apropriadas para evitá-los, resolvê-los ou mitigar seus danos, alcançando um manejo seguro. Qualquer nova tecnologia, novos medicamentos, ou novos estudos trazem muitos benefícios, mas, ao mesmo tempo, novas ameaças à segurança do paciente. A implantação de ferramentas que ofereçam subsídios para atuação na prática rotineira se mostra importante no desenvolvimento do cuidado^(5)^.

Os EAs ocorrem em frequência e intensidade diversas nos diferentes TKI. Embora não seja objetivo deste artigo classificar os EAs por medicamento, é de competência do profissional de saúde diferenciar cada um para melhor planejamento do cuidado^(2,3)^. Conhecer a frequência, a intensidade e a interferência na qualidade de vida do paciente pode ser fator determinante para planejar o manejo precoce dos pacientes mais suscetíveis.

O PRO-CTCAE™ propicia avaliar graus de medida, como gravidade do sintoma, frequência e a sua interferência na qualidade de vida. As validações de construto, confiabilidade e compreensibilidade foram exitosas^(53)^.

Usar um instrumento validado oferece informação adicional para suplementar a equipe no cuidado. O PRO-CTCAE™ proporciona uma evolução sistematizada dos sintomas, clarificando e oferecendo consistência e eficiência no registro do cuidado^(54)^.

O adoecimento na cronicidade induz os pacientes a entender seu estado de saúde, tratamento, tornando-os capazes de dizer algo sobre o que estão passando ou sentindo. O ponto crucial da equipe assistencial é decidir quais EAs serão investigados pelo PRO-CTCAE™, quer seja por via eletrônica ou impressa. Para isso, é necessário um levantamento dos EAs sobre o tratamento indicado^(55)^. É necessário, portanto, pensar em alternativas que façam do seu uso uma ferramenta útil não só nas pesquisas clínicas, mas também em qualquer planejamento do cuidado.

Para a escolha dos itens, faz-se necessário ter conhecimento prévio do que está sendo tratado. Esta foi a ação da fase 1 deste estudo, ao buscar na literatura todos os EAs de uma determinada classe medicamentosa para conseguir criar um PROM de fácil e bom uso. A revisão integrativa de literatura permitiu o embasamento teórico para a construção de uma versão inicial, direcionada para a intervenção terapêutica com fármacos TKI.

De acordo com Kluetz^(55)^, a escolha dos itens deve ser baseada na sua relevância, mas com a preocupação de reduzir a redundância das questões. Deixar o questionário mais flexível torna a ferramenta mais facilmente adaptável a diferentes doenças e tratamentos^(55)^.

Em estudo com pacientes com câncer de pulmão divididos em dois grupos (grupo com osimirtinibe e grupo controle com quimioterapia convencional), foram escolhidos 28 itens do PRO-CTCAE™ para comparação dos EAs. Verificou-se que a diarreia foi o EA mais comum no grupo com osimirtinibe, e a náusea, o vômito e a perda de apetite foram maiores no grupo da quimioterapia convencional^(45)^.

Dois estudos utilizaram um método específico para a seleção dos itens do PRO-CTCAE™ para pacientes com câncer. A escolha foi através de informações do FDA, EMA, estudos controlados randomizados, auditoria dos prontuários e entrevistas com pacientes. Os questionários foram bem aceitos e considerados úteis pelos pacientes^(46,47)^.

Em outra investigação, 28 itens do PRO-CTCAE™ foram selecionados e aplicados em pacientes de sete hospitais, com variados diagnósticos de câncer e diferentes níveis educacionais, com o objetivo de verificar a equivalência e aceitabilidade do PRO-CTCAE™ em *tablet*, sistema de voz e papel. Os resultados demonstraram que responder ao questionário não foi trabalhoso até para os indivíduos com algum grau de limitação funcional^(48)^.

A pilotagem é uma fase importante dos estudos que possuem intervenção nos seus delineamentos. Em estudo que objetivou realizar validação de construto e teste de confiabilidade dos itens do PRO-CTCAE™, sugeriu-se que a escolha dos itens para uso depende de consulta com especialistas, sugestões dos pacientes e revisão de literatura^(56)^.

Os profissionais que lidam com esses indivíduos são as pessoas que têm na sua prática o constante relato e testemunho dos EAs ocorridos com as medicações, e foram, portanto, considerados capacitados para opinar sobre a pertinência ou não dos itens escolhidos através da revisão de literatura^(56)^.

A literatura evidencia que a testagem de instrumentos tipo PROM é fundamental para validar a tradução e compreensão cultural na população usuária^(57)^. Os resultados da presente investigação foram promissores neste sentido, fortalecendo a fase posterior, que será de validação da eficácia.

Com relação a sintomas encontrados, a maior porcentagem dos sintomas não estava presente nos pacientes, o que pode significar que não são sintomas que os pacientes apresentam ou o manejo desses sintomas está eficaz e por isso não são percebidos. O sintoma urticária, que foi incluído na 2^a^ rodada da técnica Delphi por sugestão de dois juízes, foi identificado por 17 pacientes do teste piloto, mostrando que é um sintoma que merece estar incluso no instrumento, assim como o sintoma hematoma, que atingiu o mesmo número de apontamentos. No teste piloto, não se obteve nenhuma sugestão de sintomas para inclusão.

Com relação ao nível educacional dos participantes, 88,88% possuíam ensino médio ou superior, que pode sugerir uma facilidade para o processo de autoavaliação e, consequentemente, maior capacidade de comunicação com a equipe assistencial no que tange à tomada de decisão sobre seu cuidado. Esses resultados podem sugerir que mais estudos devem ser realizados para verificar a relação entre o nível de letramento da população e seu entendimento e participação no seu cuidado^(58)^.

No que tange ao entendimento das perguntas, o IVC = 1 (Tabela 5) comprova que as questões estão escritas de forma a facilitar o entendimento dos pacientes. É de responsabilidade dos profissionais de saúde promover conteúdos de fácil entendimento às populações leigas^(58)^.

O fato de já ter uma versão em português do PRO-CTCAE™ junto ao *National Cancer Institute* facilita sua utilização no Brasil, cabendo aos profissionais brasileiros somente desenvolver um uso criativo direcionado à nossa população. Isso foi conquistado nas fases de estudo relatadas neste artigo.

Para pacientes com câncer que recebem terapia antineoplásica por via oral, o conceito de autogerenciamento ganha nova perspectiva. O engajamento do paciente no seu autocuidado e na rotina diária imposta pelo plano terapêutico, incorporando novos aprendizados que incluem conhecimentos dos EAs possíveis, suas gradações e cuidados específicos, deve qualificá-lo para o autogerenciamento. Está evidenciado que o comportamento participativo e proativo do paciente interfere positivamente na sobrevida de doenças crônicas. O oposto também acontece. Nesta condição, mesmo que os profissionais se unam para oferecer o melhor cuidado, se o paciente apresentar comportamentos passivos ou não participativos, a evolução do tratamento cursa com piores desfechos^(59-61)^.

O perfil do paciente proativo inclui uma excelente adesão medicamentosa (antineoplásicos e outros), adoção de comportamentos preventivos, desde higiene oral à utilização de produtos ou qualquer outra mudança de comportamento. Complementarmente, mas não menos importante, a proatividade também deve estar expressa no automanejo emocional, na percepção das emoções novas que se juntam com os sentimentos passados e muitas vezes conflituosos^(59,60)^.

A equipe multiprofissional deve ter competências para avaliar e diagnosticar déficits do paciente, situações de risco, potencialidades e fragilidades multidimensionais, incluindo a família nesta avaliação global. A família pode ser facilitadora do processo ou, ao contrário, pode ser um motivo que esteja dificultando a adaptação do paciente nesta nova fase do tratamento. Incluir o PRO-CTCAE^®^ Estudo PROM-TKI Brasil nas consultas, conversas, orientações e criar vínculo são ações cruciais para a harmonia da relação paciente/família/enfermeiro^(61)^.

Indiscutivelmente, deve-se salientar a importância da qualidade do trabalho em equipe, com destaque para a comunicação efetiva interprofissional no cotidiano de trabalho e na criação de estratégias que promovam a discussão de casos de modo a gerar decisões compartilhadas^(62)^. Neste sentido, o paciente e a família podem ser incorporados nesta equipe.

### Limitações do estudo

O que pode ter comprometido os resultados foi a não adesão de todos os especialistas nas rodadas Delphi, embora as respostas obtidas tenham sido estatisticamente suficientes para a continuidade e veracidade do estudo. A adesão dos especialistas é um dos desafios da e-Delphi, pois pode levar à não resposta por esquecimento do prazo estabelecido ou por não terem lido as mensagens recebidas no prazo estabelecido para o envio da resposta. Outra limitação importante do presente estudo reside no desenvolvimento de outros medicamentos orais TKI que possam apresentar sinais e sintomas não contemplados nas etapas do presente estudo, configurando a necessidade futura de revisão do instrumento proposto.

### Contribuições para as áreas da enfermagem, saúde ou políticas públicas

O PRO-CTCAE^®^ Estudo PROM-TKI Brasil é o primeiro PROM específico para os EAs causados pelos TKI. O estímulo ao uso de PROM na prática assistencial oncológica é desejável, e o PRO-CTCAE^®^ Estudo PROM-TKI Brasil demonstrou ser capaz de ser compreendido pelo paciente e utilizado na prática clínica para que os profissionais de saúde tenham a correta dimensão dos sintomas e da intensidade dos mesmos na perspectiva dos próprios pacientes.

O PRO-CTCAE^®^ Estudo PROM-TKI Brasil pode favorecer a excelência assistencial por demandar estratégias de gestão rápidas e eficientes para o tratamento de EAs, bem como garantir a permanência de profissionais especialistas competentes para a avaliação e monitoramento contínuos. O instrumento pode ser uma ferramenta útil para o envolvimento do paciente e da família nos cuidados e facilitação do reconhecimento do paciente/família de EAs comuns. Salienta-se que a inclusão de perguntas abertas no instrumento permite o relato adicional de sintomas que pode ser exclusivo de um novo TKI específico. Os efeitos benéficos do PRO-CTCAE^®^ Estudo PROM-TKI Brasil na prática clínica assistencial precisam ser avaliados em estudo clínico desenhado para este fim.

## CONCLUSÕES

O PRO-CTCAE^®^ Estudo PROM-TKI Brasil pode ser utilizado na prática assistencial e em pesquisas, porque foi submetido a rigoroso processo de construção, validação por juízes especialistas e avaliação de compreensibilidade e adequabilidade pelos pacientes em uso de medicamentos do tipo TKI.
